# Risk Factors Associated with Keratoconus in an Iranian Population

**DOI:** 10.18502/jovr.v18i1.12721

**Published:** 2023-02-21

**Authors:** Hossein Mohammad-Rabei, Shahrokh Ramin, Sahar Lotfi, Hamideh Sabbaghi, Farid Karimian, Saeid Abdi, Mohammad Hasan Shahriari, Bahareh Kheiri, Kourosh Sheibani, Mohammad Ali Javadi

**Affiliations:** ^1^Ophthalmic Research Center, Research Institute for Ophthalmology and Vision Science, Shahid Beheshti University of Medical Sciences, Tehran, Iran; ^2^Department of Ophthalmology, Torfeh Eye Hospital, Shahid Beheshti University of Medical Sciences, Tehran, Iran; ^3^Department of Optometry, School of Rehabilitation, Shahid Beheshti University of Medical Sciences, Tehran, Iran; ^4^Ophthalmic Epidemiology Research Center, Research Institute for Ophthalmology and Vision Science, Shahid Beheshti University of Medical Sciences, Tehran, Iran; ^5^Department of Health Information, Technology and Management, School of Allied Medical Sciences, Shahid Beheshti University of Medical Sciences, Tehran, Iran; ^6^Basir Eye Health Research Center, Tehran, Iran

**Keywords:** Associated Factors, Iran, Keratoconus, Population

## Abstract

**Purpose:**

To determine associated factors for keratoconus (KCN) in the Iranian population.

**Methods:**

In this retrospective case-control study, 100 KCN patients and 200 age- and sex-matched individuals, who were either candidates for photorefractive keratectomy or healthy referrals from the Torfeh Eye Hospital, were included as the case and control groups, respectively. KCN patients were all registered at the Iranian National Registry of Keratoconus (KCNRegⓇ). Demographic characteristics, patients' symptoms and their habits, as well as systemic and ocular disorders were documented. Clinical examinations included best corrected visual acuity (BCVA) and refractive error measurements, biomicroscopic examination, and corneal imaging.

**Results:**

In this case group, the frequency of mild, moderate, and severe KCN was 38%, 28%, and 34%, respectively. Parental consanguinity (odds ratio [OR] = 1.758, *P* = 0.029), positive familial history in patients' first degree (OR = 12.533, *P* < 0.001) and second degree (OR = 7.52, *P* < 0.001) relatives, vernal keratoconjunctivitis (OR = 7.510, *P* = 0.003), severe eye rubbing (OR = 10.625, *P* < 0.001), and systemic diseases including migraine, hypertension, and thyroid disease (OR = 6.828, *P* = 0.021) were found as associated factors for KCN. Lesser frequency of KCN was observed in patients with Fars ethnicity (OR = 0.583, *P* = 0.042), with higher levels of wealth indices (OR = 0.31, *P* < 0.001) and higher levels of education (OR = 0.18, *P* = 0.024).

**Conclusion:**

Severe eye rubbing, vernal keratoconjunctivitis, parental consanguinity and positive familial history of KCN, low socioeconomic status, and low levels of education were significantly associated with KCN in our study population.

##  INTRODUCTION

Keratoconus (KCN) is a progressive non-inflammatory corneal thinning disorder which commonly occurs bilaterally and asymmetrically. It appears in most patients between the second and fourth decades of their lives.^[1,2,3]^ In previous literature, a prevalence of 50 to 2300 cases per 100,000 individuals has been reported for this disease in different geographical regions.^[4,5,6,7]^ The annual incidence rate of KCN was reported within the range of 22.3 to 24.9 per 100,000 population in Yazd, a province of Iran with a hot and dry climate.^[8]^ Most patients complain of reduced vision due to induced myopia and irregular astigmatism secondary to the deformation of corneal tissue into a cone-shaped structure.^[1,2,3]^ The reduction of visual acuity among patients decreases activity and quality of life.^[1,9]^

Various environmental, socioeconomic, and familial factors including atopic diseases and eye rubbing,^[10,11,12]^ exposure to sunlight and ultraviolet radiation,^[1,10]^ race,^[10]^ older age,^[10,13]^ low socioeconomic status or low levels of education,^[14]^ parental consanguinity,^[15]^ and positive familial history^[10]^ have all been reported as the probable risk factors for KCN. However, these risk factors are controversial.^[3,10,16,17,18,19]^

Considering different geographical regions, less prevalence of KCN has been found in Northern Europe, ${}^{\left[10,20,21\right]}$ North America, ${}^{\left[10,22,23\right]}$ and Japan ${}^{\left[10,24\right]}$ as compared with India, China,^[10,25,26]^ and Eastern Mediterranean countries.^[10,27,28]^

Due to the importance of KCN as a leading cause of visual impairment and the effectiveness of implementing preventive approaches in some higher risk individuals, we aimed to determine the possible associated factors for KCN among the Iranian population.

##  METHODS

### Patient Enrolment

In this case–control study, a total of 300 subjects in the age range of 16 to 61 years were studied as case (*n* = 100) and control (*n* = 200) groups between March 2016 and October 2017. KCN patients were all registered at the Iranian National Registry of Keratoconus (KCNRegⓇ). The study protocol adhered to the Declaration of Helsinki and was approved by the Ethics Committee of the Ophthalmic Research Center, Shahid Beheshti University of Medical Sciences via the approval number IR.SBMU.ORC.1397.12. The study details were explained to all participants and written consent was obtained prior to enrolling into the study.For the case group, we used the demographic and clinical records of 100 registered KCN patients from the KCNRegⓇ. The control group comprised 200 age- and sex-matched individuals who were either candidates for photorefractive keratectomy or healthy referrals from the Torfeh Eye Hospital. We excluded patients who were under suspicion of having a diagnosis of KCN or any other corneal pathological disorders from the control group.

### The Questionnaire

Data collection was performed using a standard questionnaire based on the questionnaire used by Owen and Gamble,^[29]^ which had been applied previously in other studies.^[15,28]^ In order for the questionnaire to be more comprehensive it was independently translated to the Persian language by two bilingual translators (an ophthalmologist and an optometrist). The two translators mutually agreed about any differences. The document was also translated back into English by another translator and compared to the original questionnaire to check the consistency of the translation. It was then presented to an expert panel which included ophthalmologists, biostatisticians, and research methodologists to assess its format and content validity. Finally, we asked 10 patients to read the questionnaire to determine whether there were any items that they could not understand. No shortcomings were detected by these individuals. In this questionnaire, demographic characteristics such as age, sex, occupation, birthplace, ethnicity, parental consanguinity, patients' education and socioeconomic status were questioned. Patients' symptoms (blurred vision, diplopia, and dry eye) and their habits (smoking, eye rubbing, duration of sunlight exposure, and wearing of contact lens or sunglasses) were also documented. Comorbidities of either general or systemic disorders (diabetes, atopic diseases, mitral valve prolapse, collagen diseases, renal dysfunction, and any other systemic disorders) and/or ocular diseases (vernal keratoconjunctivitis and glaucoma) among patients were also recorded in both groups. In addition, family history of KCN as well as any history of previous corneal surgery was recorded. For both the case and control groups, the questionnaires were filled either on paper, online, telephone, or via face-to-face interviews in order to increase the response rate.

### Visual and Ocular Examinations

Clinical examinations included measurements of the best corrected visual acuity (BCVA), refractive error, biomicroscopic examination, and corneal imaging using Tomey topography (TMS-4, Tomey, Nishi-Ku, Japan) and Pentacam (WaveLight Allegro Topolyzer Vario, Alcon, United States). Funduscopy was also conducted by an indirect binocular ophthalmoscope through dilated pupil using a 78D lens.

**Table 1:** Comparison of demographic characteristics between case and control groups.


orange**Factors **	orange**Level **	orange**Total**	orange**Groups**		orange**OR **	orange**95% CI**		**orange** ***P*** -value*
		orange**KCN (** ***n*** ** = 100)**	orange**Control (** ***n*** ** = 200)**	orange**Lower**	orange**Upper**	
Age (yr)	Mean ± SD	30.14 ± 7.93	29.54 ± 8.54	30.44 ± 7.61	0.985	1.017	0.955	0.356
	Median (Range)	29 (16 to 61)	28 (16 to 61)	29 (16 to 55)		
Sex	Male	150 (50.0%)	50 (50.0%)	100 (50.0%)	1	<@0.619 1.616		> 0.999
	Female	150 (50.0%)	50 (50.0%)	100 (50.0%)	1	<@		
Birthplace	North	24 (8.0%)	7 (7.0%)	17 (8.5%)	0.961	<@0.378 2.443		0933
	South	9 (3.0%)	4 (4.0%)	5 (2.5%)	1.867	<@0.483 7.207		0.365
	West	66 (22.0%)	31 (31.0%)	35 (17.5%)	2.067	<@1.164 3.671		0.013
	East	10 (3.3%)	1 (1.0%)	9 (4.5%)	0.259	<@0.032 2.094		0.205
	Center	191 (63.7%)	57 (57.0%)	134 (67.0%)	1	<@		
Patient's ethnicity	Fars	108 (36.0%)	28 (28.0%)	80 (40.0%)	0.583	0.347	0.981	0.042
	Others	192 (64.0%)	72 (72.0%)	120 (60.0%)	1	<@		
Parental consanguinity	Yes	95 (31.7%)	40 (40.0%)	55 (27.5%)	1.758	1.059	2.916	0.029
	No	205 (68.3%)	60 (60.0%)	145 (72.5%)	1	<@		
Patient's education (yr)	0–6	8 (2.7%)	5 (5.0%)	3 (1.5%)	1		
	6–12	162 (54.0%)	65 (65.0%)	97 (48.5%)	0.402	0.093	1.741	0.223
	> 12	130 (43.3%)	30 (30.0%)	100 (50.0%)	0.18	0.041	0.797	0.024
Patient's occupation	Indoor	200 (66.7%)	67 (67.0%)	133 (66.5%)	1	<@		
	Outdoor	100 (33.3%)	33 (33.0%)	67 (33.5%)	0.978	0.587	1.628	0.931
Wealth index	Rich	102 (34.0%)	19 (19.0%)	83 (41.5%)	0.31	0.165	0.583	< 0.001
	Normal	92 (30.7%)	36 (36.0%)	56 (28.0%)	0.871	0.493	1.539	0.635
	Poor	106 (35.3%)	45 (45.0%)	61 (30.5%)	1	<@		
Sunlight exposure during a day (hr)	0–2	112 (37.3%)	37 (37.0%)	75 (37.5%)	1	<@		
	3–4	88 (29.3%)	30 (30.0%)	58 (29.0%)	1.048	0.581	1.893	0.875
	5–6	34 (11.3%)	11 (11.0%)	23 (11.5%)	0.969	0.427	2.2	0.941
	> 6	66 (22.0%)	22 (22.0%)	44 (22.0%)	1.014	0.531	1.933	0.968
Wearing of sunglasses	Yes	128 (42.7%)	40 (40.0%)	88 (44.0%)	0.841	0.516	1.37	0.487
	No	172 (57.3%)	60 (60.0%)	112 (56.0%)	1	<@		
	
	
white<bcol>9</ecol>KCN, keratoconus; OR, odds ratio; CI, confidence interval; SD, standard deviation ${}^{*}$ Based on binary logistic regression

**Table 2:** Comparison of clinical characteristics between case and control groups to find the probable associated factors.


orange** Factors **	orange** Level **	orange**Total**	orange**Groups**		orange** OR **	orange**95% CI**		orangeP-value*
		orange**KCN (** ***n*** ** = 100)**	orange**Control (** ***n*** ** = 200)**	orange**Lower**	orange**Upper**	
KCN in family	First-degree	35 (11.7%)	28 (28.0%)	7 (3.5%)	12.533	<@5.21 30.148		< 0.001
	Second-degree	17 (5.7%)	12 (12.0%)	5 (2.5%)	7.52	2.54	22.21	< 0.001
	No	248 (82.7%)	60 (60.0%)	188 (94.0%)	1	<@		
Systemic diseases	Diabetes	3 (1%)	2 (2.0%)	1 (0.5%)	4.552	0.405	51.200	0.22
	MVP	6 (2.0%)	2 (2.0%)	4 (2.0%)	1.138	0.203	6.388	0.883
	Renal	3 (1.0%)	2 (2.0%)	1 (0.5%)	4.552	0.405	51.200	0.220
	Asthma	7 (2.3%)	1 (1.0%)	6 (3.0%)	0.379	0.045	3.222	0.374
	Eczema	18 (6.0%)	8 (8.0%)	10 (5.0%)	1.821	0.684	4.849	0.231
	Allergy	65 (21.7%)	21 (21.0%)	44 (22.0%)	1.086	0.593	1.988	0.789
	Others	8 (2.7%)	6 (6.0%)	2 (1.0%)	6.828	1.338	34.841	0.021
	No	190 (63.3%)	58 (58.0%)	132 (66.0%)	1	<@		
Ocular diseases	Glaucoma	17 (5.7%)	7 (7.0%)	10 (5.0%)	1.577	0.58	4.286	0.372
	VKC	13 (4.3%)	10(10.0%)	3 (1.5%)	7.510	2.015	27.997	0.003
	No	270 (90.0%)	83 (83.0 %)	187 (93.5%)	1	<@		
Eye rubbing	Never	110 (36.7%)	25 (25.0%)	85 (42.5%)	1	<@		
	Low	157 (52.3%)	50 (50.0%)	107 (53.5%)	1.589	0.909	2.776	0.104
	High	33 (11.0%)	25 (25.0%)	8 (4.0%)	10.625	4.266	26.463	< 0.001
Dry eye	Never	177 (59.0%)	53 (53.0%)	124 (62.0%)	1	<@		
	Mild	90 (30.0%)	31 (31.0%)	59 (29.5%)	1.229	0.716	2.111	0.454
	Moderate	28 (9.3%)	13 (13.0%)	15 (7.5%)	2.028	0.903	4.555	0.087
	Severe	5 (1.7%)	3 (3.0%)	2 (1.0%)	3.509	0.570	21.614	0.176
			<@		
Smoking	Yes	35 (11.7%)	7 (7.0%)	28 (14.0%)	0.46	0.193	1.093	0.079
	No	265 (88.3%)	93 (93.0%)	172 (86.0%)	1	<@		
	
	
white<bcol>9</ecol>KCN, keratoconus; OR, odds ratio; CI, confidence interval; MVP, mitral valve prolapse; VKC, vernal keratoconjunctivitis; NA, not available ${}^{*}$ Based on binary logistic regression; ${}^{**}$ Not available because of zero observation in one of the levels

**Table 3:** Comparison of topo- and tomographic findings in case and control groups.


orange** Factors **	orange**KCN (** ***n*** ** = 100)**				orange ***P*** -value*	orange**Pairwise comparison**	orange**Control (** ***n*** ** = 200)**	orange ***P*** -value**
	orange**Total**	orange**Severe (1)**	orange**Moderate (2)**	orange**Mild (3)**		
Kmin (D)	48.04 ± 4.53	50.46 ± 5.09	48.21 ± 3.23	44.63 ± 1.9	< 0.001	(1,3) (2,3)	43.24 ± 1.62	< 0.001
	47.3 (39, 65.05)	49.7 (39, 65.05)	48.15 (42.6, 57.87)	45.35 (39.8, 46.95)		43.35 (38.4, 46.7)	
Kmax (D)	51.57 ± 5.02	54.2 ± 5.71	51.93 ± 3.76	47.71 ± 1.42	< 0.001	(1,3) (2,3)	44.26 ± 1.96	< 0.001
	50.8 (40.28, 64.14)	54.2 (40.28, 62.0)	51.1 (45.63, 64.14)	47.92 (45, 49.8)		44.3 (38.8, 55.7)	
Kmeans (D)	49.81 ± 4.28	52.33 ± 4.42	50.07 ± 3.25	46.17 ± 1.54	< 0.001	(1,3) (2,3)	43.74 ± 1.67	< 0.001
	49 (40.7, 61.0)	52.35 (40.7, 59.5)	49.3 (46.75, 61.0)	46.3 (42.85, 48.3)		43.75 (38.6, 50.7)	
CCT (µm)	468.75 ± 47.89	429.26 ± 41.61	476 ± 34.79	499.56 ± 35.88	< 0.001	(1,2) (1,3)	543.32 ± 32.5	< 0.001
	469 (352, 574)	430 (352, 551)	472 (401, 539)	499 (437, 574)		541.5 (405, 623)	
	
	
white<bcol>9</ecol>KCN, keratoconus; K, keratometry; D, diopter; CCT, central corneal thickness; µm, micrometer ${}^{*}$ Based on ANOVA (Bonferroni Post Hoc Multiple comparison); ${}^{**}$ Based on independent *T*-test

**Table 4:** The stages of keratoconus and possible associated factors for severity.


orange**Factors**	orange**Level**	orange**Total**	orange**Severity of KCN (** ***n*** ** = 100)**			orange ***P*** -value*
		orange**Severe (** ***n*** ** = 38)**	orange**Moderate (** ***n*** ** = 28)**	orange**Mild (** ***n*** ** = 34)**	
Birthplace	North	7 (7.0%)	1 (2.6%)	4 (14.3%)	2 (5.9%)	0.476
	South	4 (4.0%)	1 (2.6%)	2 (7.1%)	1 (2.9%)	
	West	31 (31.0%)	13 (34.2%)	7 (25.0%)	11 (32.4%)	
	East	1 (1.0%)	0 (0.0%)	1 (3.6%)	0 (0.0%)	
	Center	57 (57.0%)	23 (60.5%)	14 (50.0%)	20 (58.8%)	
Patient's ethnicity	Fars	28 (28.0%)	11 (28.9%)	7 (25.0%)	10 (29.4%)	0.916
	Others	72 (72.0%)	27 (71.1%)	21 (75.0%)	24 (70.6%)	
Parental Consanguinity	Yes	40 (40.0%)	18 (47.4%)	12 (42.9%)	10 (29.4%)	0.280
	No	60 (60.0%)	20 (52.6%)	16 (57.1%)	24 (70.6%)	
Patient's education (yr)	0–6	5 (5.0%)	3 (7.9%)	2 (7.1%)	0 (0.0%)	0.559
	6–12	65 (65.0%)	25 (65.8%)	17 (60.7%)	23 (67.6%)	
	> 12	30 (30.0%)	10 (26.3%)	9 (32.1%)	11 (32.4%)	
Wealth index	Rich	19 (19.0%)	2 (5.3%)	5 (17.9%)	12 (35.3%)	0.019
	Normal	36 (36.0%)	14 (36.8%)	10 (35.7%)	12 (35.3%)	
	Poor	45 (45.0%)	22 (57.9%)	13 (46.4%)	10 (29.4%)	
			
KCN in family	No	60 (60.0%)	24 (63.2%)	18 (64.3%)	18 (52.9%)	0.096
	First-degree	28 (28.0%)	13 (34.2%)	7 (25.0%)	8 (23.5%)	
	Second-degree	12 (12.0%)	1 (2.6%)	3 (10.7%)	8 (23.5%)	
Ocular diseases	Glaucoma	7 (7.0%)	2 (5.3%)	4 (14.3%)	1 (2.9%)	0.826
	VKC	10 (10.0%)	4 (10.5%)	2 (7.1%)	4 (11.8%)	
	No	83 (83.0%)	32 (84.2%)	22 (78.6%)	29 (85.3%)	
Eye rubbing	Never	25 (25.0%)	8 (21.1%)	6 (21.4%)	11 (32.4%)	0.373
	Low	50 (50.0%)	19 (50.0%)	13 (46.4%)	18 (52.9%)	
	High	25 (25.0%)	11 (28.9%)	9 (32.1%)	5 (14.7%)	
	
	
white<bcol>7</ecol>KCN, keratoconus; VKC, vernal keratoconjunctivitis ${}^{*}$ Based on Chi-square test

### Definitions

KCN was diagnosed if any of the following signs were observed in any of the examination steps; presence of scissor reflex in retinoscopy, as well as Vogt's striae, Fleischer ring, Munson's sign, stromal thinning, corneal scar, and hydrops in biomicroscopy. KCN diagnosis was also determined based on the corneal topography in cases with a Sim k > 45.57D, corneal irregularity measurement (CIM) between 0.69 and 100 µm, surface regularity index (SRI) higher than 0.56, inferior–superior (I–S) asymmetry > 1.8, and KCN severity index (KSI) > 30%.^[30]^

The severity of KCN was determined based on the Rabinowitz classification^[30]^ by an experienced cornea and anterior segment specialist (HMR). Subjects were included in the control group if none of the KCN signs were observed in their examination. First-degree relatives were considered as the patients' offspring, siblings, or their parents while the second-degree relatives included their uncles, aunts, nephews, nieces, grandparents, grandchildren, half-siblings, and double cousins. We asked the patients to determine the duration of eye rubbing during a day, where it was classified as high in cases with a duration of 10 to 180 s, low with eye rubbing of < 10 s, and never in cases with no reports of eye rubbing during a day.^[10]^

### Statistical Analysis 

To present data, we used mean, standard deviation, median, range, frequency, and percentage. To evaluate the differences between the groups, we used the Fisher's Exact Test and the Chi-square Test. We also used generalized estimating equations when needed to consider the possible correlation of the results in the eyes. To assess the effect of the associated factor on the incidence of KCN, we first entered the associated factor in the model as univariate then each variable which had a *P*-value of < 0.2 was entered into the model as multivariate. All statistical analyses were performed using the SPSS software version 25 (IBM Corp. Armonk, NY: IBM Corp.). *P*-values < 0.05 were considered statistically significant.

##  RESULTS

In total 100 KCN patients and 200 controls with a mean age of 30.14 ± 7.93 years participated in the present case–control study. In the case group, the frequency of mild, moderate, and severe KCN was 38%, 28%, and 34%, respectively. Table 1 represents the comparison of baseline characteristics between the case and the control groups. As shown, no significant difference was found between the two studied groups regarding age, sex, ethnicity, occupation, sunlight exposure, and wearing of sunglasses. However, it was found that patients living in the west regions of Iran were at a higher risk of KCN as compared to the controls (odds ratio [OR] = 2.067, *P* = 0.013). Furthermore, parental consanguinity (OR = 1.758, *P* = 0.029) was also found as another associated factor for KCN. However, less frequency of KCN was observed in patients with Fars ethnicity (OR = 0.583, *P* = 0.042), higher levels of wealth indices (OR = 0.31, *P* < 0.001), and higher levels of education (OR = 0.18, *P* = 0.024).

Table 2 shows the comparison of clinical characteristics between the case and the control groups. As presented, higher risk of KCN was observed in patients who had a positive familial history in their first- (OR = 12.533, *P* < 0.001) and second-degree (OR = 7.52, *P* < 0.001) relatives, and in cases who had systemic diseases including migraine, hypertension, and thyroid disease (OR = 6.828, *P* = 0.021). In addition, patients with vernal keratoconjunctivitis (OR = 7.510, *P* = 0.003) and severe eye rubbing (OR = 10.625, *P* < 0.001) were found to be at a higher risk of KCN as compared with the healthy controls. No significant differences were observed when comparing the other factors between the two groups.

Description of topographic findings at different stages of KCN and the control group is summarized in Table 3. As shown, higher keratometric values (Kmin, Kmax, and K-means) and thinner central corneal thickness were observed in severe KCN patients as compared with the other stages of KCN and the normal controls (*P* < 0.001 for both).

Table 4 represents the effect of various factors on different stages of KCN. As shown, low wealth index was the only variable identified as associated factor among patients with severe KCN (*P* = 0.019). No statistically significant association was found when investigating other factors.

##  DISCUSSION

In the present study, parental consanguinity, positive familial history in patients' first- and second-degree relatives, vernal keratoconjunctivitis, severe eye rubbing, and systemic diseases including migraine, hypertension, and thyroid disease were found as associated factors for KCN. On the other hand, lesser frequency of KCN was observed in patients with Fars ethnicity, higher levels of wealth indices and education.

We observed that KCN is more prevalent in cases who have positive familial history of KCN. It has been reported that 14% of patients with KCN have an affected family member which could be considered as evidence for the hereditary nature of KCN, which is in line with our observation.^[31]^ Hashemi et al identified this significant association between the incidence of KCN and positive familial history.^[32]^

Furthermore, we observed that KCN patients had higher percentages of parental consanguinity, which is in line with previous studies reporting a high prevalence of KCN among populations of Pakistan, Western Mediterranean countries, and India who have a high percentage of consanguine marriages.^[10,15,33,34]^ Due to the prevalence of consanguine marriages registering as high as 40% among the Iranian population,^[35]^ more comprehensive investigations of the Iranian population might be necessary to better understand the role of inter family marriage on KCN.

In the present study, we did not detect significant associations between allergy, asthma, eczema, and KCN, however atopic diseases have been reported as one of the probable risk factors for KCN.^[10,12,32]^ This association was not detected in a study by Bawazeer et al,^[36]^ who believed that KCN is not directly related to atopic diseases. They believed, however, that it happens secondary to eye rubbing, which was also a factor significantly associated with KCN in our study. Furthermore, eye rubbing was reported as a known risk factor of KCN in a meta-analysis and systematic review conducted by Hashemi et al.^[32]^

Naderan et al^[37]^ have reported that KCN patients with vernal keratoconjunctivitis or allergic conjunctivitis have significantly thinner and steeper corneas in comparison with non-allergic KCN patients. It was concluded that these patients experience more severe KCN and should be closely followed-up and intensively treated.

Regarding the geographical location, it was found that people living in the west regions of Iran were at a higher risk of KCN. However, Kelly et al^[18]^ and Millodot et al^[38]^ showed higher prevalence of KCN among individuals living in tropical countries located around the equator, in comparison with European and North American countries. It can be considered as strong evidence of the significant association between KCN and exposure to sunlight and ultraviolet radiation. However, this significant relationship has not been found in other studies.^[16,17]^

Regarding different Iranian ethnicities, Fars people were found to be at lesser risk of KCN, which is in line with the findings reported by Hashemi et al,^[39]^ showing a higher prevalence of KCN among Arab, Turk, Kurd, and Lur ethnicities as compared to the Fars population.

Regardless of the sunlight exposure, some studies have reported less prevalence of KCN in several geographical regions like Northern Europe,^[10,20,21]^ Northern America,^[10,22,23]^ and Japan^[10,24]^ as compared to India, China,^[10,25,26]^ and Eastern Mediterranean countries.^[10,27,28]^

In addition, a strong relationship was found in children who live in families with low socioeconomic status or low levels of education,^[14]^ as children living in poor families will be dealing with problems of contaminations found in the air, water, and waste.^[40]^ We also noticed less frequency of KCN among people with higher levels of wealth indices and education.

Age is another probable associated factor with a positive relationship with the progression of KCN. Due to the incomprehensible visual symptoms in the early stages of KCN, patients are usually diagnosed in older ages with more severe stages of the disease.^[10,13][41]^

In the current study, a significant association was found between KCN and some systemic diseases including migraine, hypertension, and thyroid diseases. This finding was inconclusive due to the limited numbers of affected patients.

One of the possible drawbacks of the present study is the selection bias regarding the participation of KCN patients with different levels of wealth status.

In summary, based on our findings, severe eye rubbing, vernal keratoconjunctivitis, parental consanguinity and positive familial history of KCN, low socioeconomic status and low levels of education were significantly associated with KCN in the Iranian population.

##  Acknowledgements

This article is taken from the disease registry, titled "The Iranian National Registry of Keratoconus (KCNRegⓇ)", code number IR.SBMU.ORC.REC.1397.12 from the ethics committee, which was supported by the Deputy of Research and Technology at Shahid Beheshti University of Medical Sciences, Tehran, Iran (http://dregistry.sbmu.ac.ir).

##  Financial Support and Sponsorship

None.

##  Conflicts of Interest

The authors have no conflict of interest with the subject matter of this manuscript.
